# Focal Palatitis (Previously Focal Palatine Erosions) in Captive Cheetahs (*Acinonyx jubatus*)

**DOI:** 10.3389/fvets.2021.682150

**Published:** 2021-07-12

**Authors:** Gerhard Steenkamp, Sonja C. Boy, Paul J. van Staden, Marthán N. Bester

**Affiliations:** ^1^Institute of Mammal Research, Department of Zoology and Entomology, Faculty of Natural and Agricultural Sciences, University of Pretoria, Pretoria, South Africa; ^2^Department of Companion Animal Clinical Studies, Faculty of Veterinary Science, University of Pretoria, Pretoria, South Africa; ^3^Centre for Veterinary Wildlife Studies, Faculty of Veterinary Science, University of Pretoria, Pretoria, South Africa; ^4^Department of Oral Pathology, School of Dentistry, Sefako Makgatho Health Sciences University, Pretoria, South Africa; ^5^Lancet Laboratories, Pretoria, South Africa; ^6^Department of Statistics, Faculty of Natural and Agricultural Sciences, University of Pretoria, Pretoria, South Africa

**Keywords:** focal palatine erosions, palatitis, molar, palatal depressions, teeth, cheetah

## Abstract

Focal palatine erosion (FPE) is a misleading term that is used in the literature to describe inflammatory lesions associated with depressions of the palatal mucosa in cheetah. Cheetahs have large cheek teeth and these depressions are formed to accommodate them. Previously FPE was only described as a mandibular molar tooth malocclusion on the hard palate due to suspected rotation and super eruption of the mandibular molar teeth of cheetahs aged 18 months and older. Two hundred and fifty six cheetahs (135 male, 121 female), originating from two independent facilities, had their oral cavities evaluated as part of an annual health visit over a decade. Ninety-nine cheetahs were seen once, 59 cheetahs were seen twice, 33 were seen three times, 43 on four occasions, 16 on five occasions, 5 on six occasions, and 1 cheetah was seen seven times. Apart from these clinical cases a prospective study on 5 cheetah cubs (3 male and 2 female) was conducted to document their skull development and mandibular molar tooth eruption over a period of 25 months. Of the 261 cheetahs observed none developed rotation or super eruption of their mandibular molar teeth. The term FPE is a misnomer as these inflammatory lesions were found in palatal depressions opposing any of the cusps of all of the cheetah mandibular cheek teeth. It consisted mainly of deep ulcerations, inflammation and oedema and also micro abscess formation. In severe cases oro-nasal fistulas were present. Of all the depressions present on the cheetah's palate, the large one palatal to the 4th maxillary premolar tooth was most commonly affected. In the five cubs evaluated prospectively, focal palatitis was evident from the 7 month evaluation, before all the permanent teeth erupted. Conservative treatment of the inflamed depressions by removing the foreign material through curettage and copious flushing reduced the grade of the inflammation when observed on follow-up. Focal palatine erosion is an incorrect term used to describe focal palatitis that occurs randomly in cheetahs. This focal palatitis is often associated with foreign material trapped in the palatal depressions. Conservative management is sufficient to treat these animals without odontoplasties.

## Introduction

Focal palatine erosions (FPE) was first described in the cheetah in 1982 by Fitch and Fagan. Initially seen as a disease of captivity, it was later described to occur in both captive and wild cheetahs in Namibia (1, 2). Shortly after its introduction in the literature, an FPE study evaluation protocol was developed by the Colyer Institute (San Diego, USA) in order to quantify this clinical problem; a document to this effect is still available on their website (3). FPE lesions were previously described to occur as a result of the mandibular molar tooth that maloccluded with the palate, penetrating the palatal tissues as a result, and leading to oronasal fistula formation (1). The latter condition was evidenced clinically by “drooling” (ptyalism), “a runny nose,” “foul breath” (halitosis) and a “nasal discharge” (1, 4).

The malocclusion of the mandibular molar tooth and ensuing FPE was speculated to be the result of eating soft processed diets deficient of adequate structural composition (like bones) for optimal muscle stimulation. Reduced chewing stress was emphasized as an epidemiological factor for occlusal disorders (1), a view shared by Phillips et al. (5), and prompted a further study to evaluate the prevalence of FPE in cheetahs that consume natural prey with a high bone content (6). The results, however, challenged earlier speculations regarding diet and captivity as the only cause for FPE, and the authors (1) postulated malocclusion of the molar teeth to be of concern in the pathogenesis of these lesions (2). Subsequently, treatment of FPE in captive animals relied on the reduction of the caudal cusp of the mandibular molar tooth to reduce tooth contact with the palate, and was expected to decrease the possibility of FPE development (1, 4).

All reports of FPE in cheetahs concur it to be evident only after 10 months of age and to occur most commonly in Namibian cheetahs (1, 2, 5). A later comprehensive investigation on dry skulls, however, showed FPE to affect many other felids apart from cheetahs, the species in which it was initially described (7).

The standard anatomy of the cheetah's palate was recently described along with clear evidence of relative macrodontia of the cheetah's mandibular cheek teeth when compared to those of lions and leopards (8). The standard palatal depressions which accommodate the mandibular cheek teeth, periodically trapped foreign objects such as grass, sand or even dietary bone fragments resulting in inflammation of the mucosa and occasionally the palatal bone of these structures (8).

Apart from one case described previously (8) no other histological study describing the pathology in what was always known as FPE is available in the English literature. No pathogenic mechanisms or plausible etiologic factors, apart from malocclusion of the mandibular molar tooth (1), have been investigated to explain the development thereof. No longitudinal description of the ultimate outcome of this pathological process has been documented either.

In the present study a clinical scoring system to quantify the disease in cheetahs is introduced along with more histological analysis of these lesions. Novel hypotheses toward explaining the etiology and pathogenesis of the disease still known as FPE are proposed and a detailed overview of the clinical management of FPE in cheetahs is presented, along with the recommendation of changing the term FPE to focal palatitis (FP). The term FP will be used in this manuscript to describe any lesion, previously known as FPE, with clinical inflammation.

## Materials and Methods

Cheetahs from the captive breeding facility, the Ann van Dyk Cheetah (DW) center in South Africa (S 25° 40′ 25.1^′′^ E 27° 55′ 25.4^′′^) and the rescue sanctuary for trapped or injured wild cheetahs at AfriCat Foundation (AF) in Namibia (S 20° 51′ 59″ E 16° 38′ 22^′′^), were examined during the period 2002–2012. Cheetahs were examined as part of annual health examinations performed at these sanctuaries or when cheetahs had dental fractures. The AfriCat Foundation is housed on a farm in the Okonjima district in Namibia. The camps are large (more than 1 hectare per cheetah) and consist of sandy soil with savannah vegetation. The diet mainly consisted of pieces of meat (mainly donkey) also admixed with a mineral and vitamin supplement. The pieces of meat were presented to the cheetahs in bowls, however, the cheetahs remove the pieces of meat which then come into contact with the sand and often becomes contaminated with the sand before they consume it. The Ann van Dyk Cheetah centre is a sanctuary which houses cheetahs in small camps with very little sand. The area is on a rocky outcrop and has some grass covering as well. They are mainly fed minced chicken combined with commercial cat food together with vitamin and mineral rations fed from bowls. The facilities, cheetahs and specific data recorded by the lead researcher were previously described in detail (9). Data previously not reported include the mandibular molar tooth height, quantification of the FP using a clinical four scale scoring system, as well as a description of the content found trapped within the palatine depressions accommodating the mandibular cheek teeth (Table 1). Molar tooth height was recorded on the caudal cusp of each molar and measured with a periodontal probe from the cemento-enamel junction to the highest point of the cusp. If the molar tooth was not fully erupted this was noted and the measurement not used in any evaluations. The depth of the palatal depressions was measured with a periodontal probe from the deepest point of the depression to the palatal ridge (8). Palatal depressions were identified according to the maxillary tooth adjacent to it (e.g., dRPM4/dLPM4 referred to the large palatal depressions situated palatal to the right and left maxillary 4^th^ premolar teeth) (Table 1).

**Table 1:** The descriptive data recorded for each cheetah examined.

**Observation**	**Score**	**Criteria/Description**
Palatal depression number	dRPM2 dLPM2 dRPM3 dLPM3 dRPM4 dLPM4	Palatal to right premolar tooth 2 Palatal to left premolar tooth 2 palatal to right premolar tooth 3 Palatal to left premolar tooth 3 Palatal to right premolar tooth 4 Palatal to left premolar tooth 4
Palatal depression depth	mm	mm depth of depression
	−1	Oro-nasal fistula
Mandibular Molar height	mm	mm cemento-enamel junction (CEJ) to caudal cusp tip
	−1	Tooth absent
Clinical focal palatitis score	−1	No indentation was present
	0	Depression mucosa normal
	1a	Slight inflammation in depression, may bleed after 2 s of probing, no debris
	1b	Slight inflammation in palatal depression, may bleed after 2 s of probing, debris present
	2a	Moderate to severe inflammation, bleeds spontaneously or within 2 s of probing, no debris
	2b	Moderate to severe inflammation, bleeds spontaneously or within 2 s of probing, debris present
	3a	Oronasal fistula formed, inflammation not present, no debris in fistula
	3b	Oronasal fistula formed, Inflammation with debris present
Depression content	−5 1 2 3 4 5 6	Not Recorded Bone Grit (sand/stones) Grass Other plant material/bark Hair Feathers Other (Note)

*Included is a novel focal palatitis score as well as noting the presence and type of material present in the palatal depressions of the cheetahs*.

Five additional cheetahs originating from the Ann van Dyk Cheetah centre were evaluated clinically and radiographically from 7 months of age, and again on average every 6 months through 25 months of age. This study carried ethics approval from the University of Pretoria's animal ethics committee (Project no. EC062-11).

Any depression that presented with inflammation (focal palatitis) was manually cleared of any debris (if present) and then flushed with tap water in a 20 ml syringe. No odontoplasty or extraction of the mandibular molar teeth were done and no animal received antibiotics or anti-inflammatory drugs. A detailed histological examination of three inflamed palatine depressions were performed on 10 μm sections stained with haematoxylin and eosin. Statistical analysis, including hypothesis testing, was done using SAS software, Version 9.3 of the SAS System for Windows (SAS Institute, Cary, North Carolina, USA) and IBM SPSS Statistics for Windows, Version 23.0 (IBM Corporation, New York, USA). The 3-D column charts in **Figures 3**, **4**, **8** were drawn with Wolfram Mathematica, Version 12 (Wolfram Research, Inc., Champaign, Illinois, USA). Results were interpreted as significant when *p*-value < 0.05, and highly significant when *p*-value < 0.01.

The statistical analyses included logistic regression to estimate the prevalence of FP based on facility, sex and age of the cheetahs. Wald Chi-squared tests based on maximum likelihood estimates obtained from the fitted logistic regression model were used to test the hypotheses that, keeping other variables constant,

- the prevalence of FP is dependent on the facility,
- the prevalence of FP is different between male cheetahs and female cheetahs,
- and the prevalence of FP is affected by the age of the cheetahs.

Note that the null hypothesis for each of the abovementioned Wald Chi-square tests is that the response variable (prevalence of FP) is independent from the explanatory variable (facility, sex or age). If the null hypothesis of a Wald Chi-squared test was rejected, the particular explanatory variable was kept in the logistic regression model and the estimated odds ratio corresponding to this explanatory variable was then determined to quantify the effect of the explanatory variable on the prevalence of FP.

Correlation analyses were done between the height of the mandibular molar teeth and the palatal depression depths, as well as between the mandibular molar teeth heights and FP, and between the depth of the depressions and FP.

Firstly, by calculating Pearson's product-moment correlation coefficient and the *p*-value associated with this correlation measure, it was determined whether there is a positive correlation (i.e., positive linear relationship) between the height of the mandibular molar teeth and the depth of the depressions. A positive correlation between these two variables implies that an increase in height of the mandibular molar teeth corresponds to an increase in depth of the depressions. The null hypothesis for the hypothesis test is that the correlation coefficient is zero so that there is no correlation between these two variables.

Secondly, point biserial correlation coefficients (10) were calculated for AF cheetahs and for DW cheetahs

- between the height of the mandibular molar teeth and the presence or absence of FP,
- and between the palatal depression depths and the presence or absence of FP.

The point biserial correlation coefficient is used when one of the variables is dichotomous (i.e., categorical with only two categories). Again the null hypothesis for the test associated with this correlation statistic is that the correlation coefficient is zero, indicating no correlation between the two variables under consideration.

Finally, the distributions of the probabilities for the presence and level of FP were calculated and graphically presented to determine whether there was continuous deterioration of FP over time. Kolmogorov-Smirnov tests were used to compare the distributions of probabilities for FP across dRPM2 and dLPM2, across dRPM3 and dLPM3, and across dRPM4 and dLPM4 for AF cheetahs and for DW cheetahs, with the null hypothesis that there is no difference between the distributions of probabilities for FP across these pairs of right and left maxillary teeth.

## Results

A total of 256 cheetahs were seen at least once at AF in Namibia and DW in South Africa. The descriptive statistics of the cheetahs inspected are shown in Table 2, which also contains the number of times that individual cheetah were evaluated during the study period (2002–2012). Ninety-nine cheetahs were seen only once, 59 cheetahs were seen twice, 33 were seen on three occasions, 43 on four occasions, 16 on five occasions, five on six occasions, and one cheetah was seen seven times (Table 2).

**Table 2:** Descriptive statistics of the number of cheetahs, sex, and the number of visits per cheetah as well as per facility.

	**AfriCat**		**The Ann van Dyk Cheetah Centre**	
**Descriptive statistics**	**Male**	**Female**	**Male**	**Female**
**Number (N)**	47	45	88	76
**Age (months)**				
Mean	65.52	56.64	34.70	40.11
Standard deviation	41.35	37.17	23.85	29.27
First quartile (Q1)	30.0	24.0	15.0	15.0
Median	66.0	55.5	27.0	28.0
Third quartile (Q3)	96.0	84.0	47.5	50.0
**Number of visits**				
1	15	12	40	32
2	4	20	20	15
3	6	9	11	7
4	10	3	13	17
5	6	1	4	5
6	5	0	0	0
7	1	0	0	0

*The cheetahs' ages together with the means, medians 1st (Q1) and 3rd (Q3) quartiles are given*.

### Focal Palatitis (FP) Frequencies

**Figure 1:**
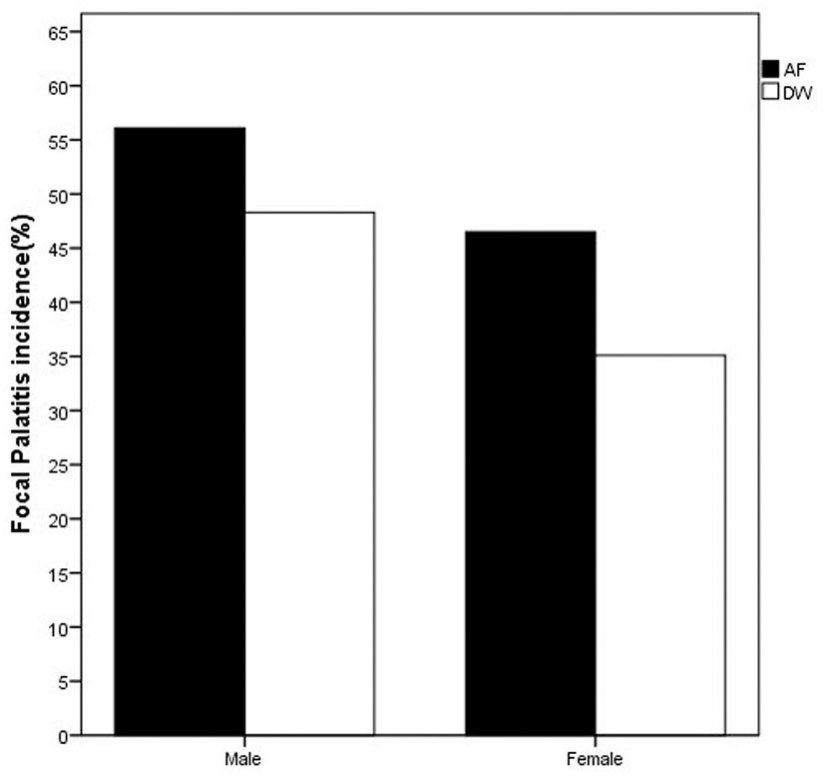
Bar chart depicting the frequency (%) of FP seen at first visit in the cheetahs of the two facilities evaluated. FP was present in cheetahs from both Namibia (AF, AfriCat) and South Africa (DW, The Ann van Dyk Cheetah Centre). Refer Table 3.

**Figure 2:**
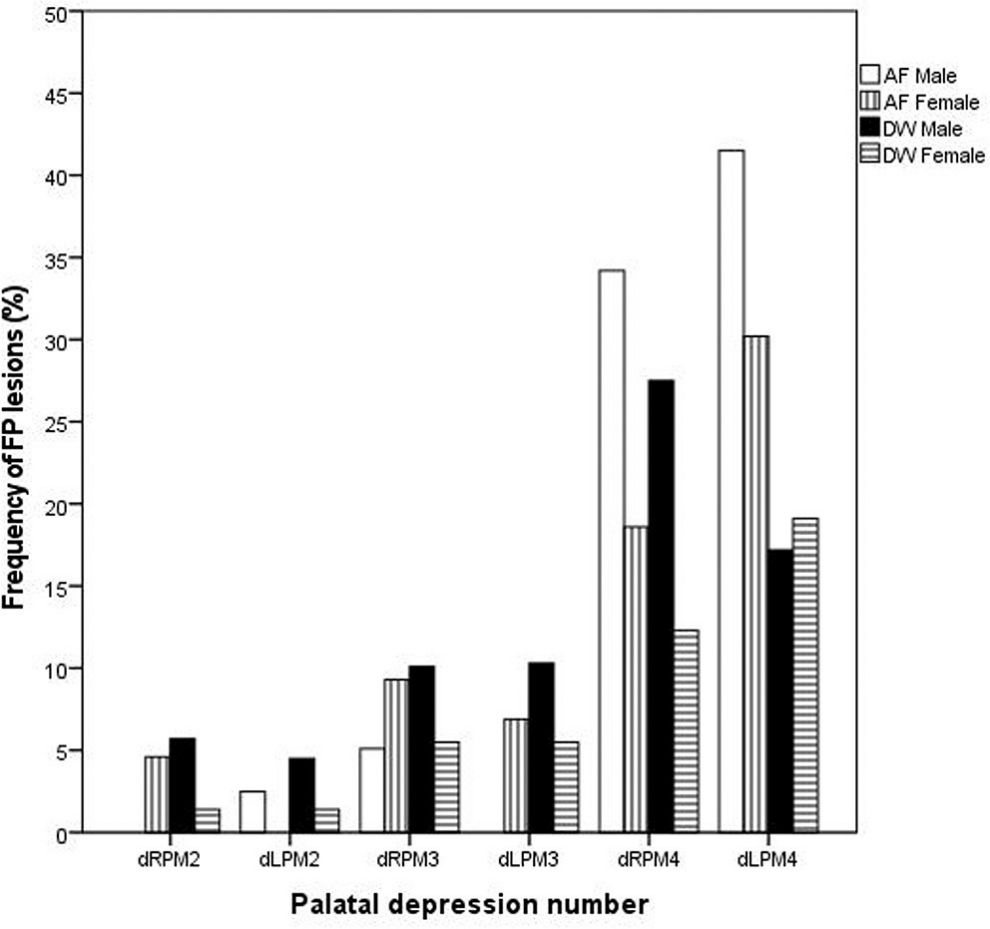
Bar chart depicting the frequency (%) of palatal depressions inflamed at the initial evaluations between the two facilities (AF, AfriCat; DW, The Ann van Dyk Cheetah Centre).

On the first evaluation, FP was seen in both sexes and at both facilities (Figure 1). The distribution of FP was similar between the two facilities with the dRPM4/dLPM4 palatine depressions most commonly affected (Figure 2).

Considering only the first visit, an analysis of the maximum likelihood estimates obtained with logistic regression together with the *p*-values of the Wald Chi-squared tests confirmed that the prevalence of FP was influenced by facility (*p*-value = 0.0233 < 0.05), sex (*p*-value = 0.0304 < 0.05) and age (*p*-value = 0.0004 < 0.01) of the cheetahs (Table 3). The odds ratio estimates corresponding to these explanatory variables are also given in Table 3. Keeping other variables constant, FP is approximately twice more likely to occur in cheetahs from AF than in cheetahs from DW (odds ratio estimate of 2.011). Also, again keeping other variables constant, the odds for male cheetahs to have FP is 81% higher than for females (odds ratio of 1/0.552 = 1.812).

**Table 3:** The influence of facility, sex and age on the prevalence of FP tested using logistic regression.

	**Maximum likelihood estimates**		**Odds ratio estimates**		
**Parameter**	**Point estimate**	***p*-value**	**Point estimate**	**95% Wald confidence limits**	
Facility (AF:DW)	0.6987	0.0233	2.011	1.100	3.679
Sex (Female: Male)	−0.5937	0.0304	0.552	0.323	0.945
Age	−0.0173	0.0004	0.983	0.973	0.992

*For each of these explanatory variables, the maximum likelihood estimates, the p-values for the Wald Chi-squared tests for independence, the odds ratio estimates and their respective 95% Wald confidence limits are given (AF, AfriCat; DW, The Ann van Dyk Cheetah Centre)*.

In order to study the relation between the heights of mandibular molar teeth and the palatal depression depths as well as their potential impact on FP, we compared the data collected from all visits, but excluded mandibular molar tooth heights and palatal depression depths equal to zero. The mean mandibular molar tooth height (n = 1002) was 10.91 mm with a standard deviation (SD) of 1.02 mm, while the mean palatal depression depth (n = 1002) was 10.38 mm (SD = 1.71 mm).

A Pearson product-moment correlation coefficient of *r* = 0.2754 (*p*-value < 0.0001) was observed between the heights of the mandibular molar teeth and the palatal depression depths. Since *r* > 0, the conclusion is that an increase in mandibular molar heights corresponds to an increase in palatal depression depths.

The point biserial correlation coefficient between the height of the mandibular molar teeth and the presence or absence of FP was *r*_pb_ = 0.0701 (*p*-value = 0.1934) for AF cheetahs and *r*_pb_ = 0.0386 (*p*-value = 0.3163) for DW cheetahs. Although the point biserial correlation coefficients were positive for both facilities, these coefficients are close to zero, implying that the height of the mandibular molar teeth are not significantly correlated with the presence or absence of FP in cheetahs in either of these facilities.

In order to establish the contribution of palatal depression depth to FP, point biserial correlation coefficients between depression depth and the presence or absence of FP were calculated for both AF and DW cheetahs. For AF cheetahs, *r*_pb_ = 0.0818 (*p*-value = 0.1100), whereas for cheetahs from DW, *r*_pb_ = 0.1056 (*p*-value = 0.0067). These results suggest that there is only a significant relation between the palatal depression depth and the presence or absence of FP for cheetahs from DW, but not for cheetahs from AF. This potential relationship between palatal depression depth and FP was further examined with logistic regression modeling. A logistic regression model using facility and depression depth to explain the presence or absence of FP was fitted. As indicated by the *p*-value from the relevant Wald Chi-squared test, FP was influenced by palatal depression depth (*p*-value = 0.0024 < 0.01). Keeping facility constant, the estimated odds ratio for depression depth was 1.147 with a 95 % Wald confidence interval of (1.050, 1.253). Therefore, although statistically significant, we note the lower limit of the 95% Wald confidence interval to be very close to 1.

**Figure 3:**
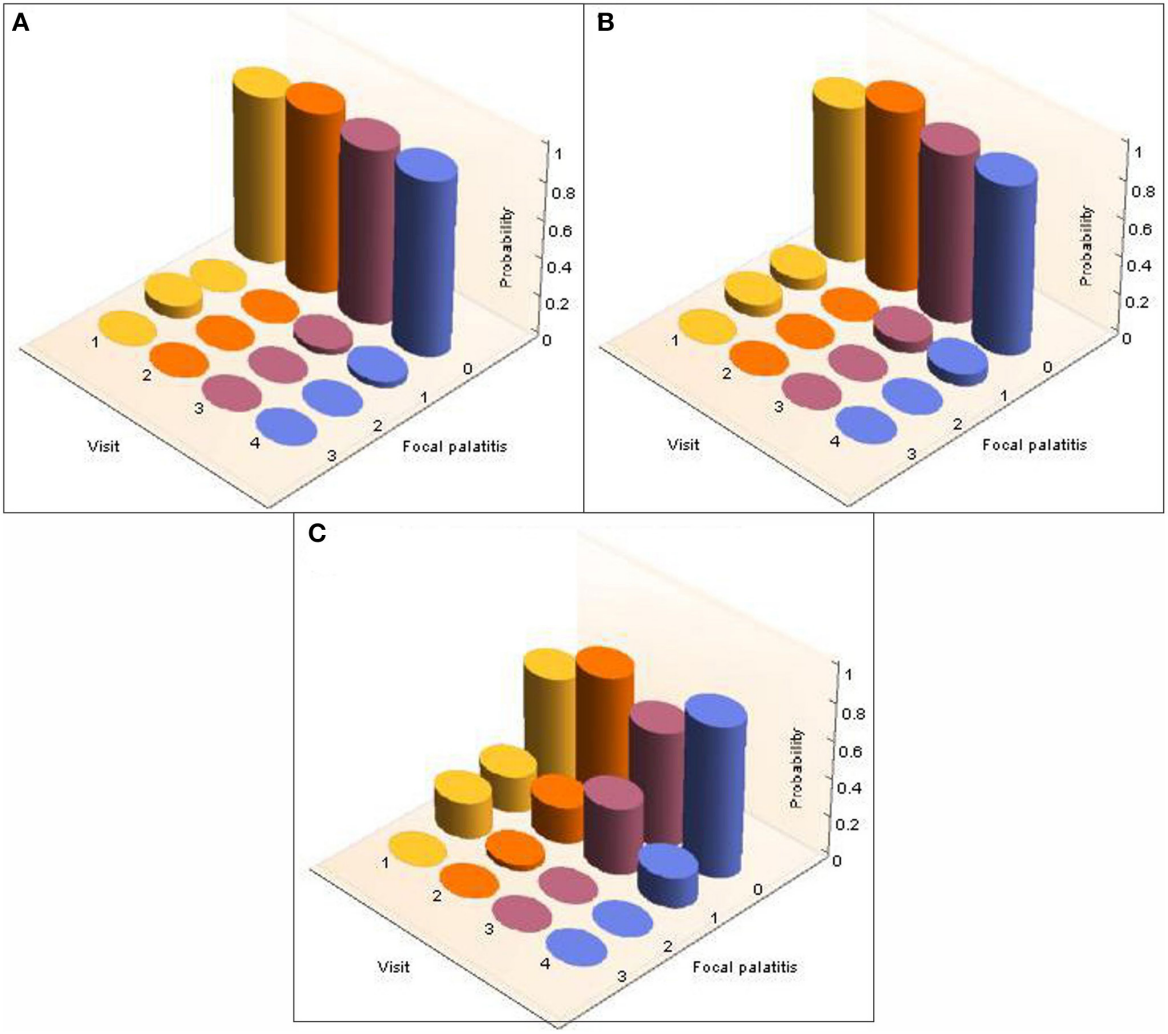
3-D column charts depicting the development of FP in terms of occurrence and severity in cheetahs from AfriCat (AF), evaluated on four different, consecutive visits. The probabilities of the development of FP in each of the palatal depressions that can potentially have FP are shown. Note that dRPM2 and dLPM2, dRPM3 and dLPM3, and dRPM4 and dLPM4 were pooled. **(A)** Shows FP at the depressions palatal to the maxillary 2nd premolar teeth, **(B)** shows FP at the depressions palatal to the 3rd maxillary premolar teeth, **(C)** shows FP at the depressions palatal to the 4th maxillary premolar teeth.

**Figure 4:**
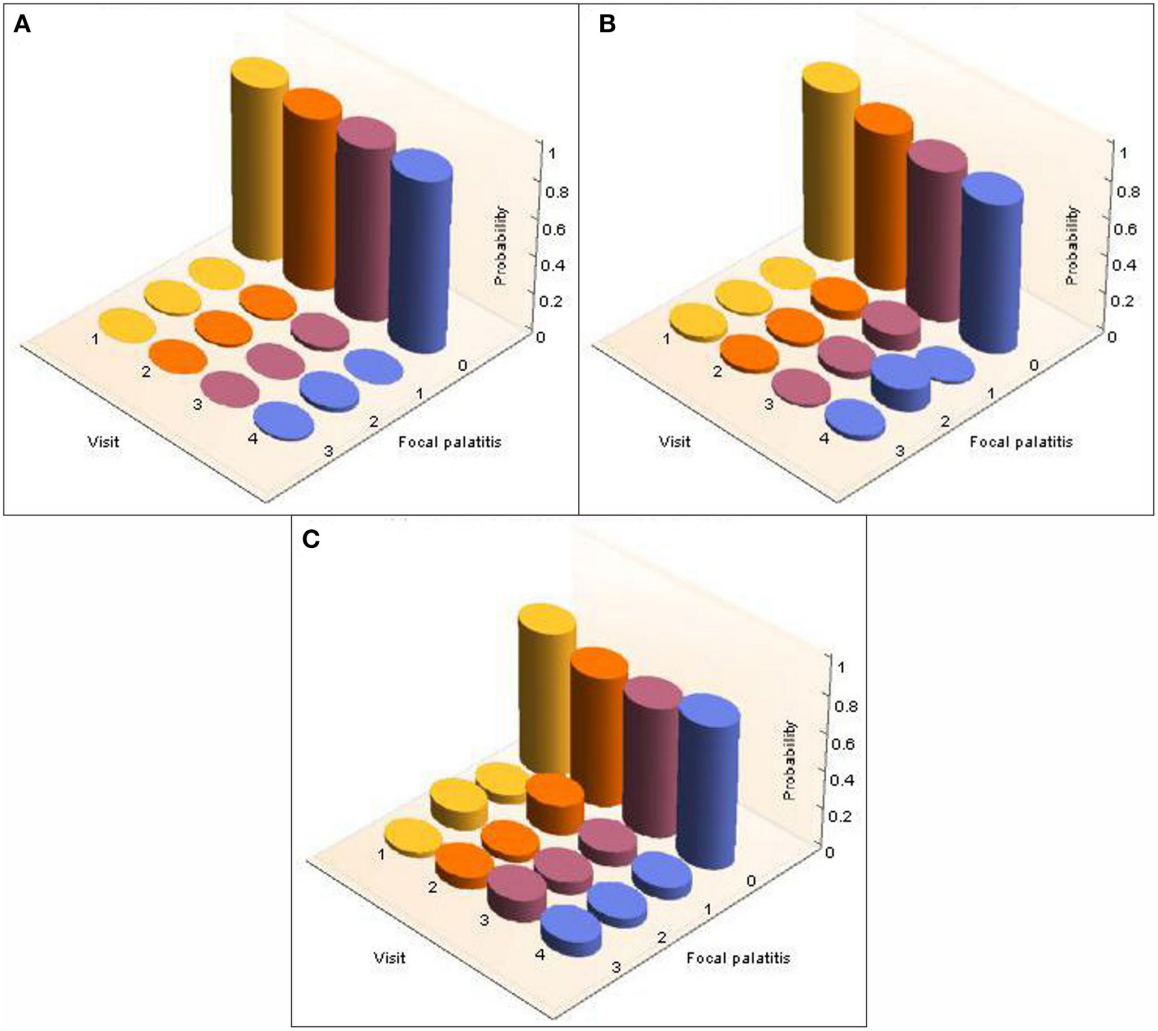
3-D column charts depicting the development of FP in terms of occurrence and severity in cheetahs from the Ann van Dyk Cheetah Centre (DW), seen on four different yet consecutive visits. The figures show the probabilities for FP in each of the palatal depressions that can potentially have FP. Note that dRPM2 and dLPM2, dRPM3 and dLPM3, and dRPM4 and dLPM4 were pooled. **(A)** Shows FP at the depressions palatal to the maxillary 2nd premolar teeth, **(B)** shows FP at the depressions palatal to the 3rd maxillary premolar teeth, **(C)** shows FP at the depressions palatal to the 4th maxillary premolar teeth.

A total of 26 cheetahs at AF and 39 cheetahs at DW were seen on four different, yet consecutive occasions (Table 2). The presence and level of FP together with the corresponding probabilities were calculated and are illustrated in Figure 3 for AF and Figure 4 for DW. Because the Kolmogorov-Smirnov tests indicated that there were no statistical differences between the distributions of probabilities for FP across dRPM2 and dLPM2, across dRPM3 and dLPM3, or across dRPM4 and dLPM4 (Table 4), the corresponding frequencies were pooled. There was no continuous deterioration of FP over time. In fact, FP appeared sporadic and may increase or decrease from one visit to another (Figures 3, 4).

**Table 4:** Comparisons of the distributions of probabilities for FP across dRPM2 and dLPM2, across dRPM3 and dLPM3, and across dRPM4 and dLPM4 for cheetahs from AfriCat (AF), from the Ann van Dyk Cheetah Centre (DW), and for the 5 additional cheetah cubs.

**Parameter**	**AF cheetah**	**DW cheetah**	**5 cheetah cubs**
dRPM2 and dLPM2	0.9705	0.7185	1.0000
dRPM3 and dLPM3	1.0000	0.3512	0.9476
dRPM4 and dLPM4	1.0000	1.0000	1.0000

*For each comparison the p-value obtained using a Kolmogorov-Smirnov test is given*.

**Figure 5:**
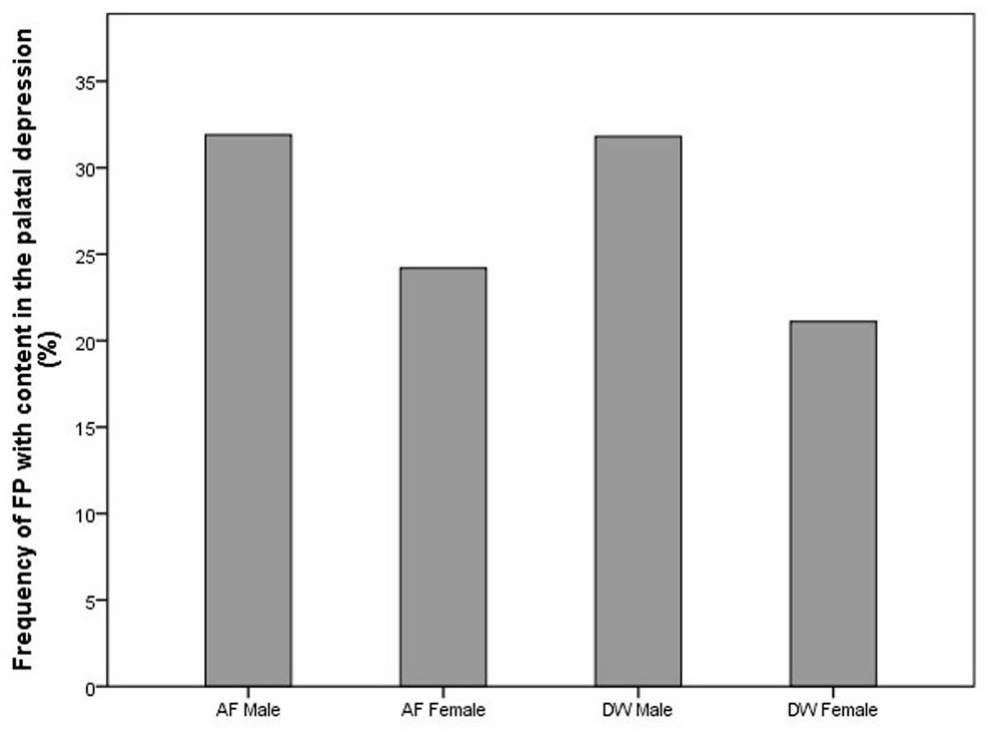
Bar chart showing the frequency (%) of content found in the palatal depressions with FP in the cheetahs of two different facilities (AF, AfriCat; DW, The Ann van Dyk Cheetah Centre).

**Figure 6:**
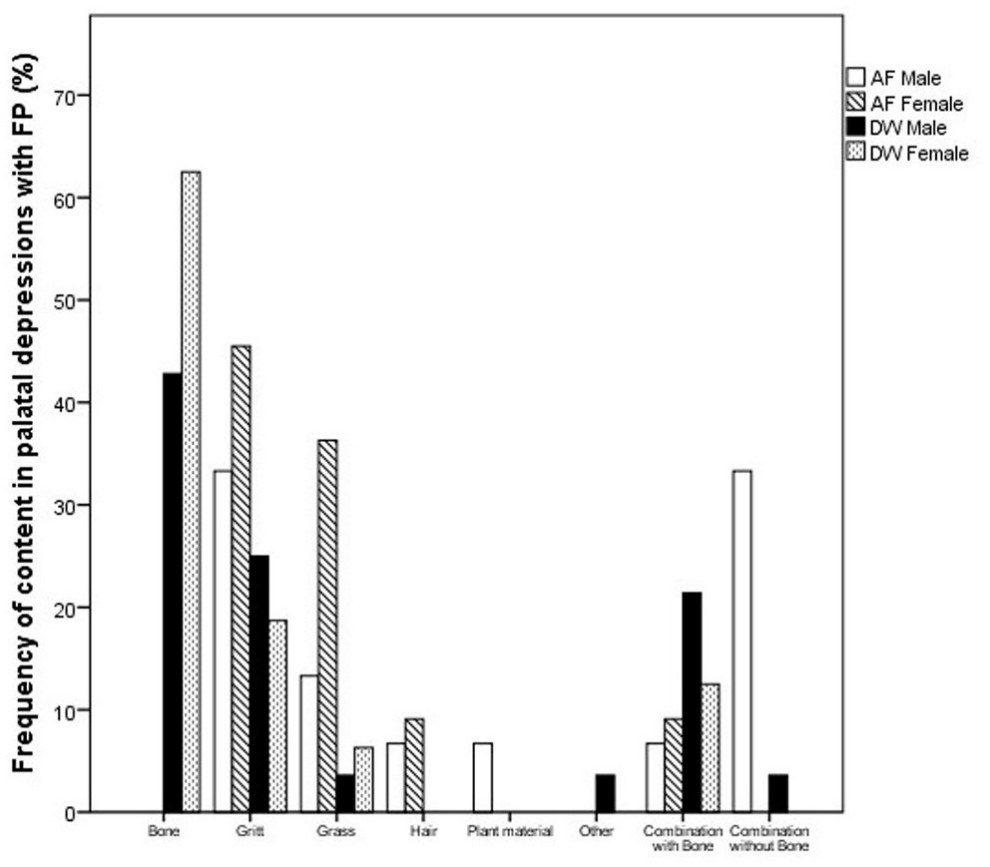
Bar chart depicting the frequency (%) of different material found in the palatal depressions of cheetahs with FP between the two facilities (AF, AfriCat; DW, The Ann van Dyk Cheetah Centre).

**Figure 7:**
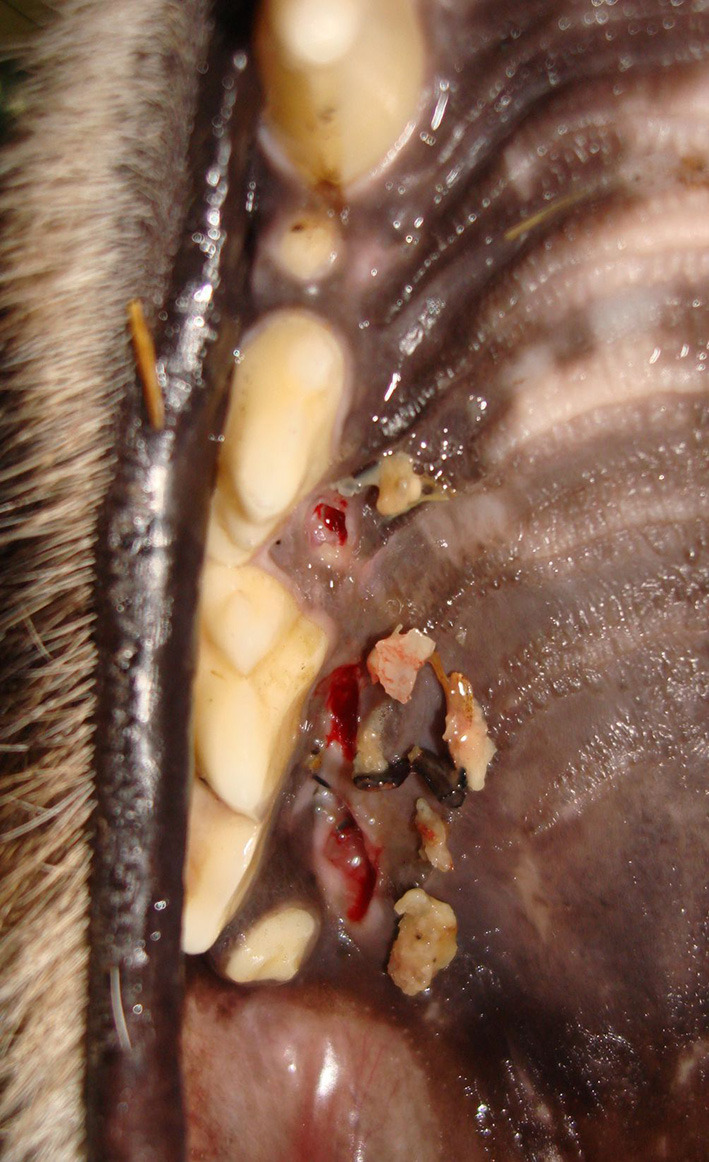
Photograph depicting foreign material such as bone fragments that were frequently encountered in the FP lesions in the cheetahs from the Ann van Dyk Cheetah Centre (DW).

Cheetahs with FP frequently had foreign material impacted in the palatal depressions (Figure 5). There is a clear distinction between the type of content and facility (Figure 6). AF showed a high frequency of grit (small sand particles) whereas at DW a very high proportion of the cheetahs had combinations of bone and other foreign material in the palatal depressions (Figure 7).

### Focal Palatitis in the Additional Cheetah Cubs

**Figure 8:**
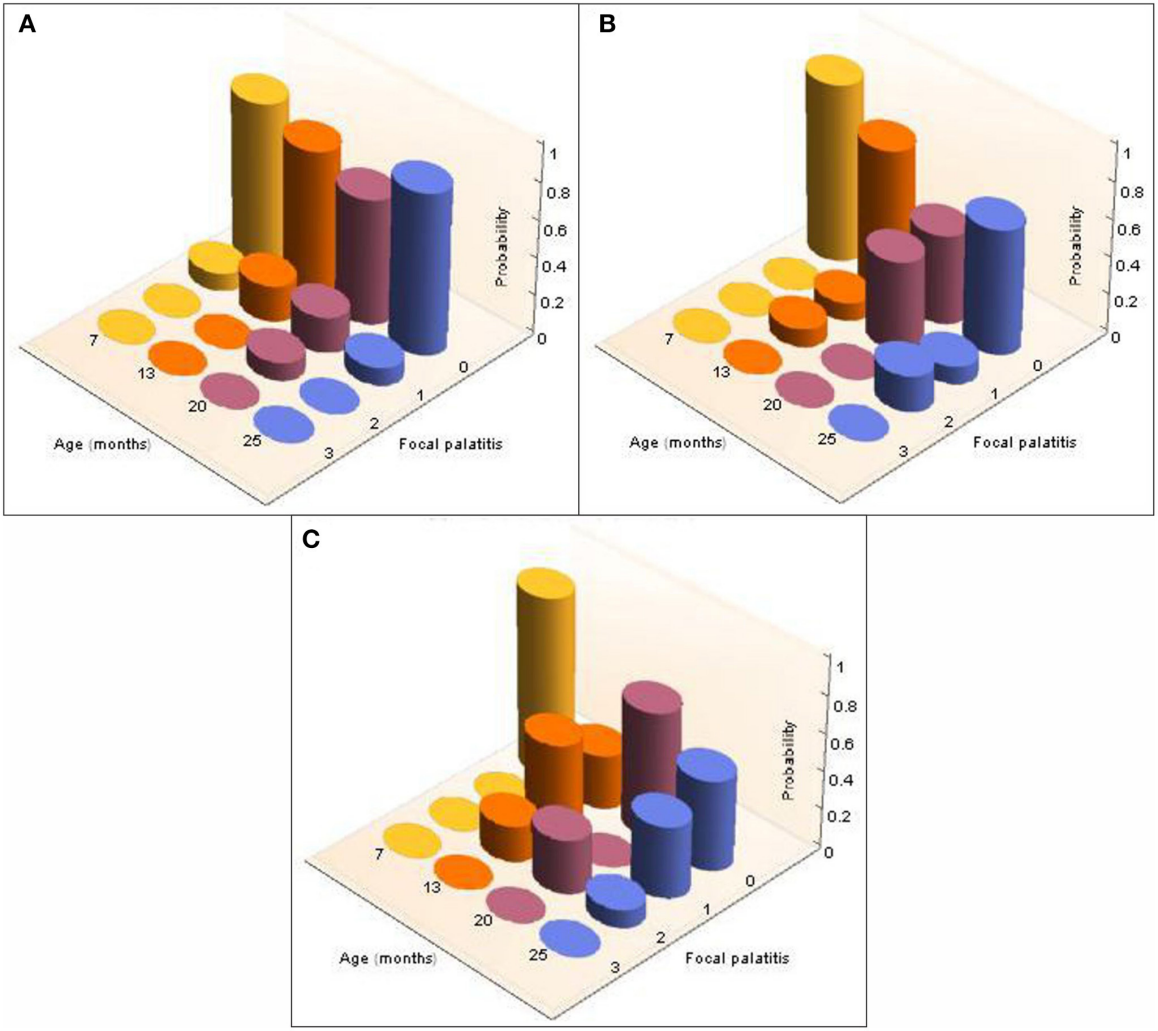
3-D column charts depicting the longitudinal development, and severity of, FP in the five cheetah cubs from the Ann van Dyk cheetah centre followed from 7 months to 25 months of age. The probabilities of finding FP present in each of the palatal depressions that can potentially have FP are indicated as a function of age (months). Note that dRPM2 and dLPM2, dRPM3 and dLPM3, and dRPM4 and dLPM4 were pooled. **(A)** Shows FP at the depressions palatal to the maxillary 2nd premolar teeth, **(B)** shows FP at the depressions palatal to the 3rd maxillary premolar teeth, **(C)** shows FP at the depressions palatal to the 4th maxillary premolar teeth.

Considering the five additional cheetah cubs, there were again no differences indicated by the Kolmogorov-Smirnov tests between the distributions of probabilities for FP across dRPM2 and dLPM2, across dRPM3 and dLPM3, or across dRPM4 and dLPM4 (Table 4). The corresponding frequencies were again pooled. FP developed in one of the five cheetah cubs at 7 months of age (M673 – dLPM2). From 13 months all five cheetahs had FP in various depressions of varying scores (Figure 8). The presence and severity of FP did not continuously increase as the cheetahs aged. The molar tooth heights did not increase once fully erupted (Table 5).

**Table 5:** The molar height (mm) of five young growing cheetahs from the Ann van Dyk Cheetah Centre (from 13 to 25 months-of-age) after eruption (0 mm at 7 months indicate these teeth were not erupted).

	**Age (months)**							
	**7**		**13**		**20**		**25**	
**Molar**	**Left**	**Right**	**Left**	**Right**	**Left**	**Right**	**Left**	**Right**
M661	0	0	12	12	12	12	12	12
M673	0	0	12	12	12	12	12	12
M668	0	0	11	11	12	12	11	12
F662	0	0	12	12	12	12	11	11
F669	0	0	11	10	11	11	10	10

Of the depressions that had inflammation with content, grit was most commonly seen impacted in these depressions (40%) (6/15) followed by pieces of dietary bone (33.3%) (5/15) and grass (26.7%) (4/15).

### Histological Description

**Figure 9:**
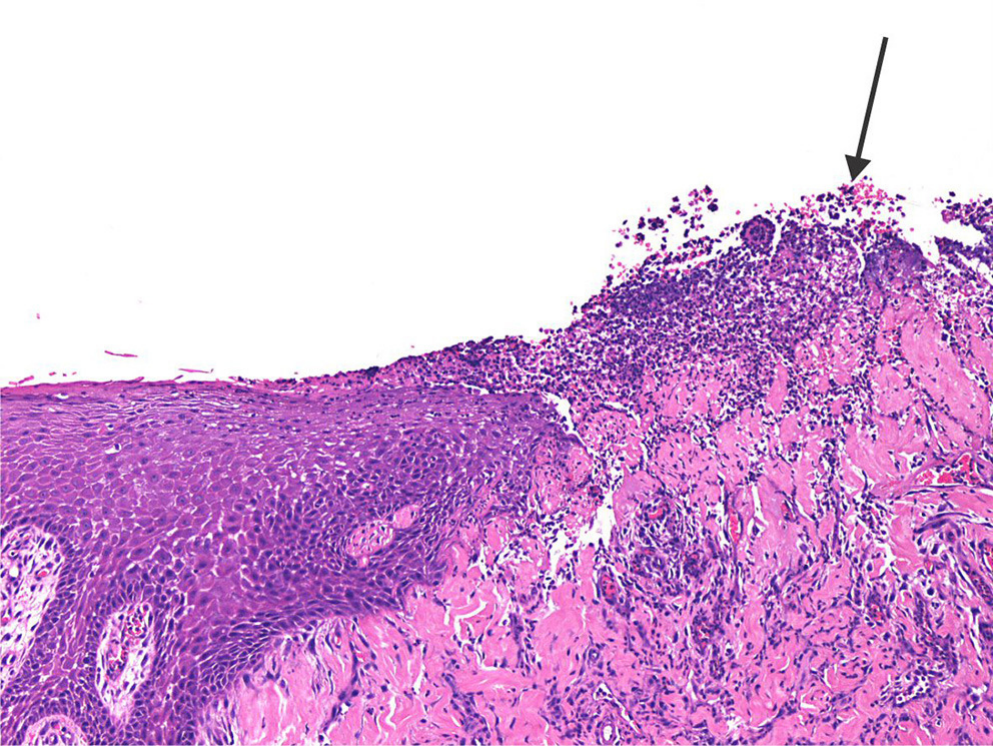
The photomicrograph shows ulcerated epithelium (arrow) with mixed inflammation extending deep into the underlying connective tissue of the inflamed palatal depression.

The three clinically inflamed depressions showed obvious ulceration of the covering epithelium and various degrees of mixed inflammation and oedema, some even showing features of micro-abscess formation with bacterial colonies extending deep into the underlying connective tissue of the depression (Figure 9). In one case foreign material in the form of plant-like structures were seen associated with the inflamed granulation tissue.

## Discussion

The bone and soft tissue depressions present in particular areas on adult cheetahs' palates were previously described as a functional structure to accommodate the relatively large mandibular cheek teeth (8). Previously termed FPE, the results of that study proved most palatal depressions to be normal anatomical structures lined by squamous epithelium to accommodate the opposing mandibular tooth cusps. Some cases presenting with clinical signs of inflammations, i.e., erythema, oedema, ulceration or even draining purulent material, usually contained foreign debris stuck deep inside the narrowest aspect of the palatal depression. We propose the term focal palatitis (FP) to be used as diagnostic/descriptive term in these latter cases. This study concludes FP to be a random event that may affect any of the previously described palatal depressions. There appears to be no longitudinal deterioration of the inflammation and even cases where small oro-nasal communication was present (FP score 3) resolved completely after conservative treatment only (Figure 4). The authors maintain that FP is a chance event and depending on the diet, may occur at any stage. Although FP (previously known as FPE) was believed to be a condition affecting Namibian cheetahs almost exclusively (1, 2, 5), this study proved it not to be the case.

The depth of the depression in the palate is a function of the height of the molar tooth, the latter remaining constant with no super eruption over time (present study). Although molar tooth height did not influence the frequency of FP in cheetahs from either facility, palatal depression depth in the cheetah at DW did. This significant association between palatal depression depth and FP at DW could probably be explained by the fact that bone fragments from the chickens fed to the cheetahs predominated in the palatal depressions at DW. These bony spicules, sharp and irregular compared to the rounded structure of the sand at AF, get trapped easier in a deep or narrow palatal depression. By contrast, AF relies more on feeding donkey meat, cut into 2–3 kg chunks for some animals and further divided into small blocks for those cheetahs that had lost a large number of teeth. Although some of these pieces of meat contain large pieces of bone, it is not consumed by the cheetahs as they are too big. Both facilities use adequate supplementation in order to assure a balanced diet.

It is clear that FP can occur from any age when the permanent mandibular cheek teeth have erupted as was seen in one seven month old cheetah cub (this study). This proves FP to occur in association with the cusps of any of the erupted permanent mandibular cheek teeth at any time (8). FP is twice as likely to occur in cheetahs housed at AF compared to those at DW, most probably as a result of the animals frequently dragging their food through the sand before eating it, resulting in sand particles often being pushed into the palatal indentations. The small but significantly higher incidence of FP in males compared to females may be the result of a higher biting force in males with foreign matter being pushed deeper and harder into the palatal indentations.

During the 18 months we evaluated the five developing cheetah cubs at DW, no change in the molar height was observed once the teeth were fully erupted. This together with our previous study that confirmed no change in angulation of the fully erupted molars (11) does not support the original hypothesis that super eruption of the molar teeth is the cause of (then FPE) FP (1).

Cheetahs at AF frequently also contained hair in the palatal depressions, more so than at DW. Although grooming in the cheetah is observed less frequently than in other big cats, during the annual evaluations at AF we often found them to have unkempt coats compared to the lions and leopards. Cheetah flies (*Hippobosca longipennis*) are bloodsucking ectoparasites that live preferentially in the fur of wild carnivores like cheetahs (12). This was a significant problem at AF, not encountered at DW. The AF cheetahs frequently bit themselves and it is speculated that this could be due to skin irritation caused by large numbers of flies present on their coats.

The researchers are aware that FP has previously been described in other Felids based on dry skulls (7). The criteria used in this study to document the presence of FPE was penetration of the palatine bone medial to the maxillary 4th premolar teeth. It is important to note that FP may cause perforation of the palatine bone in the deep aspect of the palatal depression, but that this is not the most common presentation (this study). From previous work (8) together with this study the presence of depressions to accommodate cheek teeth in cheetahs have been described. There are no such detailed descriptions available for other Felid species. Although depressions have been seen in lions and leopards palatal to the maxillary 4th premolar teeth, these are shallow (8). Once there are detailed studies available on other Felids it would be interesting to see if they have FP and to what causes it. Apart from FP several conditions like foreign bodies, osteomyelitis, trauma, palatal tumors etc. may also cause palatal bone loss and the authors therefore caution the interpretation of FP studies based only on dry skull evaluations.

The present study confirms sporadic inflammation in the palatal depressions of cheetahs, with or without the presence of foreign material. The authors hypothesize that when a normal palatal depression gets inflamed due to entrapment of any of the previously mentioned foreign materials, the inflammation will be alleviated and disappear as soon as the material is dislodged. However, if the foreign material persists, the ensuing chronic inflammation will lead to increased osteoclastic activity (13) with destruction of associated palatal bone which may even result in oro-nasal fistula formation. Focal palatitis may also clear after thorough cleaning, however, the same depression may have the same risk as before to trap foreign material again. This study proposes that the term focal palatine erosions (FPE) be changed to focal palatitis (FP), which gives a more accurate description of the disease process. Erosion refers only to a loss of some superficial epithelial cells, whilst complete loss of the epithelial covering with breach of the basal lamina is more appropriately termed ulceration. In the case of FP, the epithelium is usually ulcerated with or without underlying bone involvement and perforation. Erosion is therefore an inaccurate term to use.

None of the animals in this study was ever treated by molar tooth reduction as previously described (1). Based on the experience of treating 256 cheetahs over a 10-year period, we advocate regular cleaning of the palatal depressions with copious rinsing of inflamed depressions, another good reason for annual oral health evaluations in captive cheetahs. Molar tooth reduction should be avoided.

We conclude that FP is an inflammatory condition affecting cheetahs originating from both Namibia and South Africa. The DW cheetahs were all captive bred, while the Namibian cheetahs comprised of mainly adult animals removed from farmland as “problem animals” (9). This proves that cheetahs of any background may develop FP. These inflammatory lesions occur randomly, although the large depression opposite the mandibular molar is most commonly affected. Diet may affect FP in captive cheetahs, and FP can occur at any time when the permanent mandibular premolar or molar teeth have erupted and corresponding palatal depressions have formed. Treatment of FP should be conservative by cleaning the palatal depressions of any debris accumulated in it and copious lavage. Molar reduction should be avoided. Future studies should explore FP in wild cheetahs using larger sample sizes to evaluate the effect of sex on the prevalence of FP.

## Data Availability Statement

The raw data supporting the conclusions of this article will be made available by the authors, without undue reservation.

## Ethics Statement

The animal study was reviewed and approved by Animal Ethics Committee, University of Pretoria (Project nr. EC062-11).

## Author Contributions

GS: design of project, acquisition and analysis, interpretation, writing, and editing of manuscript. SB: design of project, acquisition and analysis, histological analyses, writing, and editing of manuscript. PvS: design of project, statistical analyses, interpretation, writing, and editing of manuscript. MB: design of project, writing, and editing of manuscript. All authors contributed to the article and approved the submitted version.

## Conflict of Interest

The authors declare that the research was conducted in the absence of any commercial or financial relationships that could be construed as a potential conflict of interest.
